# *Eryngium* Species as a Potential Ally for Treating Metabolic Syndrome and Diabetes

**DOI:** 10.3389/fnut.2022.878306

**Published:** 2022-05-20

**Authors:** Eréndira Patricia Pérez-Muñoz, Marilena Antunes-Ricardo, Mariana Martínez-Ávila, Daniel Guajardo-Flores

**Affiliations:** ^1^Tecnológico de Monterrey, Escuela de Ingeniería y Ciencias, Monterrey, Mexico; ^2^Tecnológico de Monterrey, The Institute for Obesity Research, Monterrey, Mexico

**Keywords:** diabetes, metabolic syndrome, *Eryngium*, dyslipidemia, medicinal plant

## Abstract

Medicinal plants possess natural compounds that can be used as an alternative for synthetic medicines that may cause long-term side effects on patients such as neurocognitive effects, muscular and hepatic toxicity. Metabolic Syndrome is associated with increased risk of several diseases such as diabetes, cardiovascular disease, dyslipidemia, and hypertension thus, becoming the greatest challenge as a growing public health concern worldwide. Latin-American countries possess a wide diversity of medicinal plants that have been used to treat different health conditions since pre-Hispanic times. *Eryngium* spp. has been studied due to their beneficial properties mainly to treat diabetes, dyslipidemia, blood pressure, and digestive problems. This review gives an update mainly on the pharmacological activities of the *Eryngium* spp., summarizing the biological activities and plausible mechanism of action of their bioactive components toward metabolic syndrome. For instance, flavonoids and tannins proved to increase the levels of HDL and reduced the levels of VLDL, LDL. On the other hand, phenolic acids improved glucose metabolism through the inhibition of phosphoenolpyruvate carboxykinase and glucose 6-phosphatase (G6Pase) and reestablished the impaired activity of enzymes related to glucose oxidation and glycogen synthesis. The terpenes and sesquiterpenes like β-farnese, β-pinene, and calamenene exhibited a protective effect by reducing the oxidizing damage by the regulation of the Reactive Oxygen Species (ROS). Saponins controlled the dyslipidemia by reducing the serum concentrations of lipids, triglycerides and total cholesterol. Finally, the aerial parts of *Eryngium* had the capacity of inhibiting dietary carbohydrate digestive enzymes, thus controlling glucose levels. The *Eryngium* plant is normally consumed as an infusion to obtain the benefits of the plants, however novel technologies such as cavitation, ultrasound assisted, microwave assisted, and supercritical fluid have been applied to improve the extraction yields and selectivity of bioactive compounds. The common treatment to control diabetic dyslipidemia are synthetic medicines such as metformin and ezetimibe, which allows the regulation of glucose, cholesterol and insulin resistance. However, patients that take these medications may present side effects such as muscular toxicity, hepatic toxicity, neurocognitive effects, just to name a few. More studies regarding the efficacy and safety of the use of traditional medicinal herbs are required. However, these materials may be used in the treatment of diabetes related conditions to ensure life quality and reduce side effects among the diabetic population

## Introduction

Metabolic syndrome (MS) is classified as the main cause of death according to the World Health Organization (WHO) and the Global Health Observatory (1). MS worldwide prevalence has increased around 30%, increasing the risk of morbidity and mortality about 3 times higher in comparison with a healthy population (2). MS is defined as a state of chronic low-grade inflammation due to a complex interplay between genetic and environmental factors. MS is interconnected with physiological, biochemical, clinical, and metabolic factors that directly increase the manifestation of a group of diseases including atherosclerotic cardiovascular disease, and type 2 diabetes mellitus (T2D). Among these factors are insulin resistance, visceral adiposity, atherogenic dyslipidemia, endothelial dysfunction, genetic susceptibility, elevated blood pressure, hypercoagulable state, and chronic stress (3).

Diabetes Mellitus is a metabolic disease that is associated with disorders in the metabolism of carbohydrates, proteins, and lipids that affect insulin action (4). The past two decades have seen a growing trend toward the prevalence of diabetes, being 2.8% in 2000, and it is estimated that it will reach 5.4% by 2025 (5). Approximately 220 million people suffer from diabetes and it is expected this number increases to 366 million by 2030 (6). The effects of diabetes include long-term damage, dysfunction, and failure of various organs that can lead to death. People with diabetes present 2 to 4-fold higher risk of developing cardiovascular disease than those people without it. The cardiovascular complications attributable to atherosclerosis are responsible for 70–80% of patients with diabetes. T2D is also related to obesity as a consequence of insulin resistance and hyperglycemia, being the sixth leading cause of death causing 1.6 million deaths worldwide (6). A resistance to insulin or the inefficient production of this hormone can also lead to the accumulation of lipids (e.g., dyslipidemia) as free fatty acids (FFAs) in blood and muscular tissue. The abundance of FFAs in the plasma gives place to a reduction in the insulin-regulated glucose metabolism (7). An excess of FFAs produces lipotoxicity and ectopic lipid deposition, as well as incomplete FFAs oxidation which induces the production of reactive oxygen species (ROS) and toxic lipid intermediates, thus leading to oxidative stress (8).

Thus, the main goal of the treatments against diabetes is not only the good glycemic control but also the prevention of macrovascular complications (e.g., myocardial infarction, heart failure), and microvascular complications (e.g., retinopathy, nephropathy, neuropathy) (9). Additionally, extensive research has shown the effect of the accumulation of lipids in blood and muscle tissue and the impact on cardiovascular diseases, thus a treatment that leads to the reduction and control of these lipids is essential. Statins, Ezetimibe and Metformin are the most popular treatments against T2D available in the market. However, these drugs may cause considerable side effects in patients. Around 10–25% of the patients that use statins, reported myalgia, which is one of the most frequent statin-associated side effects and it is usually the result of statin non-adherence (10). Other side effects that statins can cause in T2D patients are neurological and neurocognitive effects, hepatotoxicity; the severity of these side effects mainly depends on factors such as age, gender, severity of the diabetes (11). Ezetimibe is the second most popular treatment against T2D in combination with statins. Ezetimibe is used to reduce LDL levels, however it has been seen that it can cause liver toxicity when it is used as a monotherapy when patients are intolerant with statins (12). Another popular treatment is Metformin which is used to reduce high sugar levels, it has been reported that about 20–30% of the patients develop gastrointestinal problems. Metformin minor side effects are nausea, abdominal floating, flatulence, vomiting, diarrhea, headache, dizziness, loss of appetite, abdominal cramps, to name just a few (13).

Thus, one of the greatest challenges is to find an efficient alternative to treat metabolic syndrome and diabetes impairments. For this reason, there is currently an increasing interest in identifying ingredients from natural origin that have health benefits toward metabolic syndrome and T2D. There is a growing body of literature that recognizes the importance of some plants for medicinal purposes. On this matter, Mexico is the fourth place worldwide of countries with more medicinal plants (7), a pre-Hispanic heritage that nowadays is still in use by many indigenous communities but is at risk of being perished in the new global economy (9). One of the plants used to treat cardiovascular diseases and diabetes is popularly called “Frog grass” and belongs to the *Eryngium* spp. family. The aerial parts of this plant are consumed as an infusion and have traditionally been used to treat diabetes, dyslipidemia, blood pressure, and digestive problems (10). Species of this genus contain secondary metabolites such as flavonoids, saponins, rosmarinic acid, triterpenes, coumarins, polyacetylenes, and essential oils that give them their medicinal properties (5). For instance, characterization of the hydroethanolic extract of *Eryngium* spp. has been studied to determine its hypolipidemic effects on diabetic induced rats (11, 12). Even though it has been confirmed the hypolipidemic effects of *Eryngium* spp., most of these experiments lack traceability of the species used and therefore the effect between different species is unclear. Thus, testing the hypolipidemic effect of a specific *Eryngium* in clinical trials may help validate the use of this plant as an adjuvant in the treatment of metabolic syndrome-related diseases. The present review describes the *Eryngium* species as a viable alternative of natural treatment against diabetic dyslipidemia either as a medicine or a nutraceutical. Special attention to the oxidative stress, insulin resistance, and lipotoxicity are addressed.

## Literature Review

### Metabolic Syndrome and Pathophysiology

**Figure 1:**
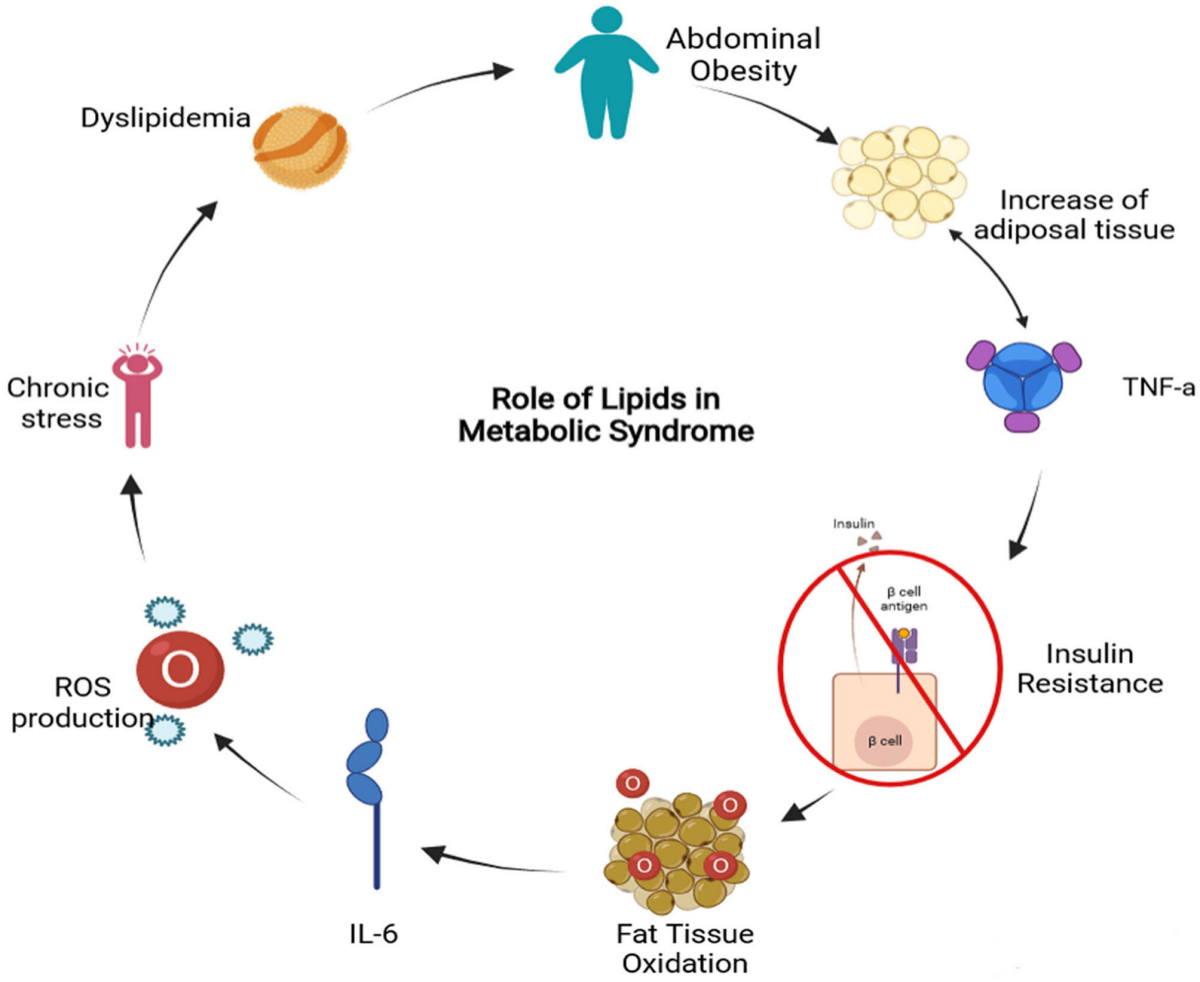
Metabolic Syndrome related to lipids diagram (TNF-a, Tumor Necrosis Factor alpha; IL-6, Interleukin-6; ROS, Reactive Oxygen Species) (Created in BioRender).

It is well-known that MS is interconnected with several physiological, biochemical, clinical, and metabolic factors that directly increase the risk of T2D and atherosclerotic cardiovascular disease. The metabolic syndrome involves chronic low-grade inflammation in which insulin resistance, visceral adiposity, atherogenic dyslipidemia, endothelial dysfunction, genetic susceptibility, elevated blood pressure, hypercoagulable state, and chronic stress are intrinsically related (14). Even though the causes of MS can be diverse, lipids play a fundamental role in a sort of ways summarized in Figure 1. Dyslipidemia is related to the activities of atherogenic lipoproteins and antiatherogenic, high-density lipoprotein C (HDL-C), which involves the increase of triglycerides, low-density lipoprotein (LDL), and lipoproteins (15). Also, the lipid accumulation in the abdominal region causes the enlargement of adipocytes reducing the oxygen supply by blood, causing hypoxia (16). Moreover, insulin resistance is caused by the inadequate response of insulin in the peripheral tissue, such as adipose, muscle, and liver (17). This results in an increase of glucose in the bloodstream that is subsequently transported to the muscle tissue and induces fatty oxidation (18). Furthermore, FFAs induce insulin resistance by the inhibition of insulin-mediated glucose uptake (19). As a result, the enzyme activity involved in the fatty acid synthesis is increased promoting the secretion of lipoproteins and inducing the gluconeogenic pathway (20). Finally, the expression of cytokines may downregulate the metabolic signaling pathways involved in MS (7). For instance, the interleukin 6 (IL-6) can modulate the inflammation process and suppress the lipoprotein lipase activity related to insulin and T2D development. The increased levels of proinflammatory cytokines also induce the production of reactive oxygen species (ROS) that can lead to cardiovascular complications (21). On the other hand, tumor necrosis factor-alpha (TNF-α) induces adipocyte apoptosis and promotes insulin resistance by the inhibition of insulin receptor substrate 1 signaling pathway.

### Metabolic Syndrome and Dyslipidemia

Dyslipidemia can be the consequence of increased production of very-low-density lipoprotein (VLDL) and LDL and a decreased HDL-C. Several factors lead to dyslipidemia and at the same time, it can lead to Type 2 diabetes and cardiovascular disease. In fact, those people that suffer from metabolic syndrome have seven times more probability of becoming diabetic and more chances of developing cardiovascular disease (22). Insulin resistance is the primary cause of Type 2 Diabetes Mellitus and is associated with dyslipidemia by the increased hepatic VLDL secretion. This also leads to overproduction of VLDL particles, which plays an important role in the formation of LDL that involves the transfer of triglycerides from VLDL to LDL by protein cholesteryl ester transfer protein (CETP) which is the substrate for the hepatic lipase responsible for increasing lipolysis of triglyceride-rich LDL (23). During metabolic syndrome, chylomicrons tend to accumulate in the circulation thereby influencing overall lipid and lipoprotein turnover. The assemblage of chylomicrons is dependent on apoB-48 and MTP. Dietary fatty acids that are carried through the circulatory system by the chylomicrons are taken up by the liver as chylomicron remnants. The accumulation of those remnants leads to impaired glucose and lipid metabolism. It has been observed that patients that suffer from both, diabetes and metabolic syndrome, have a delay in the clearance of triglycerides and chylomicrons (24). The overproduction of triglyceride-rich lipoproteins in insulin-resistant patients is mainly driven by FFAs and inflammatory cytokines. Besides the fatty acids and triglycerides, the availability of cholesterol in the intestine also influences chylomicron assembly (23).

### Type 2 Diabetes and Dyslipidemia

Diabetic Dyslipidemia among patients with Type 2 Diabetes Mellitus is very common, having a prevalence of 72-85%. This phenomenon is associated with a substantially increased risk of cardiovascular disease in comparison with healthy people. Diabetic dyslipidemia plays a central role in the genesis and the progression of atherosclerosis. The main quantitative lipoprotein abnormalities of diabetic dyslipidemia are increased triglycerides and reduced HDL-C. As well the patients have an increase in large very low-density lipoprotein subfraction (VLDL1) and small-dense LDL-C particles, which are susceptible to oxidation as well as increased triglycerides content both in LDL-C and HDL particles, and glycation of apolipoproteins. Such lipoprotein aberrations are frequently associated with insulin resistance, which may affect the activity of lipoprotein lipase, cholesteryl ester transfer protein, phospholipid transfer protein, endothelial lipase, and hepatic lipase. Diabetic Dyslipidemia is strongly related to insulin resistance, visceral obesity and non-alcoholic fatty liver disease, as well insulin resistance is associated with excessive fatty acid flux to the liver that leads to VLDL overproduction. Insulin fails to suppress lipolysis and FoxO1 which is a transcription factor involved in the regulation of gluconeogenesis and glycogenolysis by insulin signalization and it also regulates adipogenesis negatively. Diabetes not only is the cause of cardiovascular disease deaths, but also is the cause of diabetic retinopathy, lower limb amputations, and chronic kidney disease (diabetic nephropathy) (21).

### Insulin Resistance, Oxidative Stress and Lipotoxic Effects

ROS are normally generated by the cell in their metabolism. However, an excess of these molecules due to their chemical reactivity can damage macromolecules such as lipids, proteins, and nucleic acids. Due to the high reactivity of ROS, cells have defense mechanisms that regulate the production of ROS and avoid any kind of cell damage within the cell. In T2D, ROS promotes inflammation by increasing the levels of proinflammatory cytokines and the expression of cellular adhesion molecules and growth and it can lead to lipotoxicity (21). Lipotoxicity refers to the inhibition of the pancreatic β-cells due to lipid overload of the pancreatic islets (25). T2D patients are unable to respond adequately to insulin, this leads to the increase of FFAs in the blood due to the interruption of the antilipolytic effect of insulin on adipocytes. Large amounts of FFAs are released to the bloodstream, which leads to the initiation of systemic lipotoxic effects such as lipid deposition and the interruption of insulin signaling (8).

### Reactive Oxygen Species (ROS) Mechanism of Action in Diabetes

The byproducts of normal mitochondrial metabolism generate potentially damaging levels of ROS (26). These species oversee several hyperglycemia-induced pathogenic mechanisms, such as the inhibition of the enzyme glyceraldehyde-3-phosphate dehydrogenase *via* the activation of the enzyme poly-ADP-ribose polymerase-1 (PARP-1). This enzyme is involved in DNA repair and cell apoptosis pathways, and it is activated by the induction of strand breaks in nuclear DNA through the action of ROS. At the same time, the activation of PARP-1 leads to the inhibition of Glyceraldehyde-3-Phosphate Dehydrogenase (GAPDH) by poly-ADP ribosylation. Accumulation of GAPDH in cells is involved in the activation of the Advanced Glycation End products (AGEs) pathway by dragging GAPDH facilitates diacylglycerol production which activates the Protein Kinase C (PKC) pathway. Furthermore, if fructose-6-phosphate (F6P) levels are elevated, flux through the hexosamine pathway increases, where F6P is converted to UDP-N-acetylglucosamine by the action of glutamine-fructose-6 phosphate aminotransferase (GFAT). This leads to the obstruction of GAPDH and thus to the accumulation of glucose, which enhances the flux *via* polyol pathways and the consumption of Nicotinamide Adenine Dinucleotide Phosphate (NADPH) in the process (26). The polyol pathway uses NADPH for Glutathione regeneration, and when AGEs bind to their receptor, they lead to the formation of ROS (27). In the AGEs pathway, ROS are induced *via* de AGE receptor binding, leading to the activation of PKC along with the activation of nuclear factor κB (NF- κB) and NADPH oxidase, causing morbidity in mitogen-activated protein kinase (MAPK) signaling (26). In the Hexosamine pathway, under normal conditions, when glucose is at normal levels, a small amount of F6P moves away from glycolysis. However, in diabetes there is the phenomenon of hyperglycemia, in these conditions a greater amount of F6P is eliminated from glycolysis to facilitate the GFAT substrate, which allows the conversion of F6P into glucosamine-6-phosphate (G6P). Because mitochondrial ROS are elevated due to diabetes, ROS inhibit the activity of GAPDH, this enzyme is crucial for regulating NADPH levels, resulting in the accumulation of glycolytic intermediates (26). PKC activation plays an important role in T2D progression *via* vascular cell dysfunction, since it is associated with vasoconstriction, proliferation and overgrowth of smooth muscle cells. PKC also mediates barriers to gene expression of key proteins, resulting in reduced blood flow, inflammation, occlusion of capillaries and generation of free radicals that lead to damage of cellular macromolecules. PKC-dependent activation of NADPH oxidase can lead to the stimulation of ROS production due to high glucose levels. The generation of ROS mediated by NADPH oxidase tends to develop nephropathy in diabetic patients (26).

Furthermore, in people with diabetes, insulin signaling is significantly affected by ROS. Under healthy conditions, ROS is essential for proper insulin signaling. However, in diabetic patients, there is an elevation in ROS, causing a malicious effect on insulin signaling. Insulin receptor stimulation is found in adipocytes, which regulate peroxide production *via* NADPH oxidase. The rise in insulin triggers the shift in the P13-kinase signaling pathway. This abrupt P13-kinase signaling aggravates NOX4 activity, leading to phosphorylation of Rac (Rac GTPase) instead of PIP2, leading to an elevation of ROS in the cell. Elevated ROS activate casein kinase-2 CK2, triggering retromer activation. The retromer is in charge of signaling the trans-Golgi network, resulting in the transportation of GLUT4 to lysosomes for transport to the plasma membrane. This signaling process contributes to an elevated glucose level in the intravascular system, leading to a condition of oxidative stress (8). ROS promote inflammation by increasing the levels of proinflammatory cytokines and the expression of cell adhesion molecules and growth factors, leading to cardiovascular complications. The relationship between ROS and lipotoxicity is because free fatty acids are oxidized in mitochondria by β-oxidation. Increasing free fatty acid levels leads to incomplete free fat oxidation, generation and increase of ROS and toxic lipid intermediates. Due to the altered mitochondria, the oxidation of free fatty acids takes place in the endoplasmic reticulum, which causes stress in the endoplasmic reticulum (8). In other words, chronic oxidative stress leads to insulin resistance, dyslipidemia, β-cell dysfunction, glucose intolerance and T2D. Prolonged oxidative stress, hyperglycemia and dyslipidemia are detrimental to β-cell. Impaired β-cell function leads to insufficient insulin production, impaired glucose-stimulated insulin secretion, fasting hyperglycemia and development of T2D (28).

### NPC1L1 and ABCG5/ABCG8 Protein Complexes

Cholesterol homeostasis is maintained in three (3) main ways: de novo synthesis, intestinal absorption and biliary fecal excretion; these processes are mainly maintained by a variety of enzymes or protein complexes. One of these proteins is called Niemann- PickC1-Like (NPC1L1), which is in charge to mediate intestinal cholesterol absorption and biliary cholesterol reabsorption (29). NPC1L1 once is bonded with cholesterol, the NPC1L1/cholesterol complex is internalized by joining AP2 clathrinid, which creates a vesicle complex that translocate with the help of myosin along microfilaments in the cytosol to a storage endosome called endocytic recycling compartment. This phenomenon occurs when intracellular cholesterol becomes low, NPC1L1 is released from the endocytic recycling compartment and traffics back along microfilaments to the cell membrane (30).

The other protein complex is the heterodimer formed by the ATP-binding cassette transporters G5 and G8 (ABCG5/ABCG8). The ABCG5/ABCG8 has been observed to inhibit the absorption of cholesterol from the diet by mediating the efflux of these sterols from enterocytes back into the gut lumen, this mechanism occurs by promoting efficient secretion of cholesterol from hepatocytes into bile. It is worth mentioning that ABCG5/ABCG8 is at higher levels than NPC1L1 as the heterodimer is overexpressed by the presence of bile salts, thus increasing the cholesterol secretion (29). Furthermore, NPC1L1 is mainly expressed in the small intestine, and liver, while ABCG5/ABCG8 is mainly expressed in both liver and small intestine, but also in the gallbladder (31).

**Figure 2:**
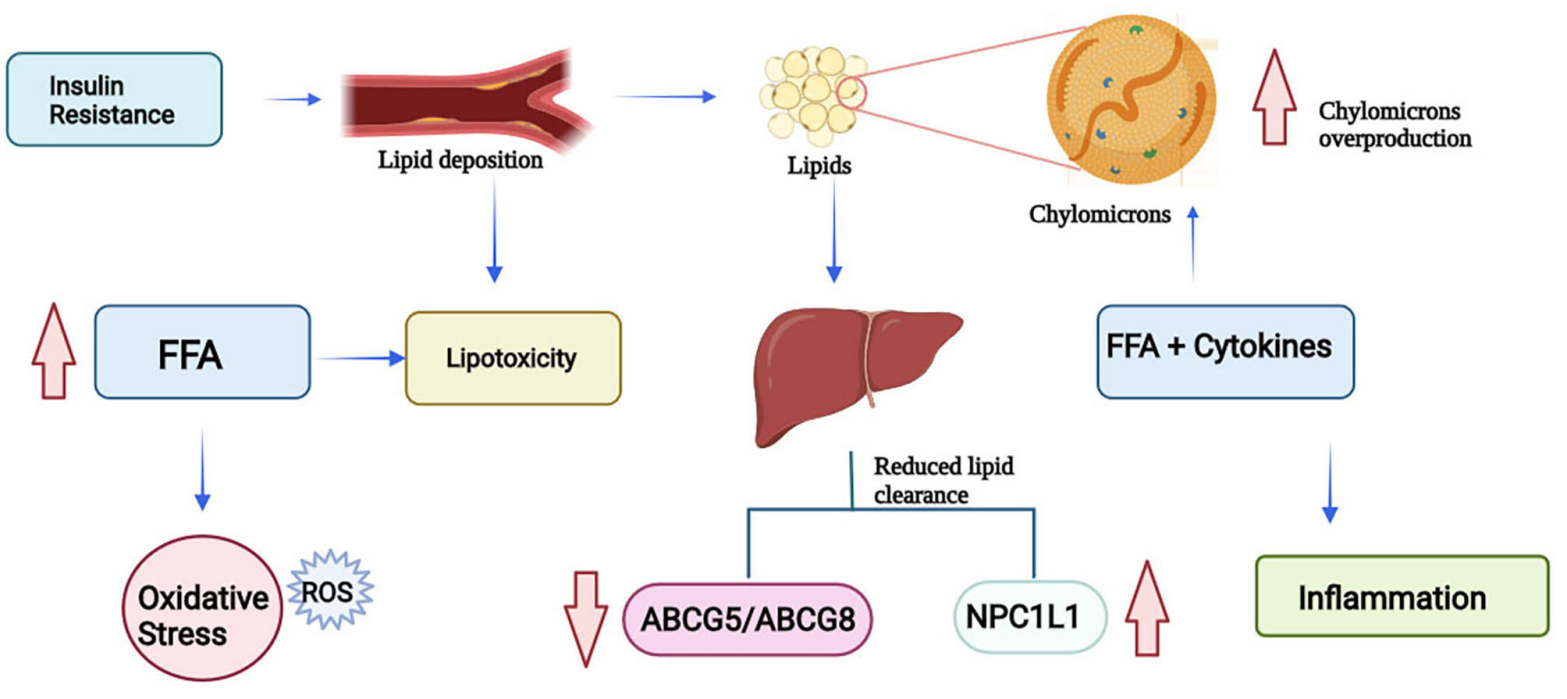
Lipotoxicity process due to insulin resistance (FFA, Free Fatty Acids; ROS, Reactive Oxygen Species; NPC1L1, Niemann-Pick C1-Like 1; ABCG5, ATP-Binding Cassette Subfamily G Member 5; ABCG8, ATP-Binding Cassette Subfamily G Member 8) (Created in BioRender).

As mentioned previously, due to insulin resistance there is a tendency to accumulate lipids and have a delayed clearance of triglycerides and chylomicrons, particularly chylomicrons which contribute to the large triglyceride-rich lipoproteins. Dietary fatty acids, after entering the circulation through chylomicrons, are taken to the liver by chylomicron remnants. The accumulation of chylomicrons leads to impaired glucose and lipid metabolism. Overproduction of triglyceride-rich lipoproteins in patients with insulin resistance is driven by FFAs and inflammatory cytokines. Besides FFAs and triglycerides, the availability of cholesterol in the intestine influences chylomicron assembly. In T2D patients it has been observed NPC1L1 levels are increased, and ABCG5/ABCG8 expression levels are reduced. By watching these levels and based on what was discussed in previous paragraphs it is safe to infer that diabetic patients have higher amounts of intestinal cholesterol available for the synthesis and secretion of chylomicrons and thus delaying the clearance process (23). This process can be summarized in Figure 2.

### Treatments Against Dyslipidemia in Both Metabolic Syndrome and Type 2 Diabetes

As discussed previously, MS is not just one illness alone, but a group of illnesses and most of those illnesses end in atherosclerotic cardiovascular disease, however, the main risk factors are abdominal obesity, and insulin resistance (32). Some of the medicines used to treat illnesses that are consequences of metabolic syndrome are Enalapril®, Captopril®, Metformin®, Statins®, and Ezetimibe®. Enalapril® and Captopril® are mainly used to treat hypertension but it has been reported that they can have side effects such as cough, increased serum creatinine, headache, and skin rash (33). Metformin® is used to treat T2D patients to regulate the glucose levels and insulin resistance (34) it has been reported that because ABCG5/ABCG8 facilitate hepatobiliary transport of cholesterol, metformin can induce an increase in the heterodimer to facilitate the disposal of excess cholesterol through the hepatobiliary *via* (35). However, a previous study of Metformin® reported that it can lead to lactic acidosis, hemolytic anemia, and pancreatitis (36). Another therapeutic approach is the use of Statins® and Ezetimibe® to regulate high levels of cholesterol in the blood. Ezetimibe® blocks the NPC1L1 protein in the jejunal brush border, reducing the uptake of intestinal lumen micelles into the enterocyte, thus preventing the formation of NPC1L1 (37). However, the main side effects are muscular toxicity, hepatotoxicity, renal toxicity, and neurocognitive effects (11). Due to the side effects of the popular prescribed medicines, most physicians and the scientific community agree that the use of herbal medicine against metabolic syndrome could lead to less stress to the organism, and that would avoid the side effects and toxicity of the chemical medicine. Also, it has been observed that the use of this type of therapy has led to equal or better results than the chemically produced medicaments, as well the socioeconomic advantage of using this type of traditional medicine (38).

### *Eryngium* Species as a Potential Ally

The species *Eryngium* consists of approximately 250 species, and are distributed in Eurasia, North Africa, North and South America. In the new world, several species of *Eryngium* are used as medicine by indigenous populations, particularly for treating digestive problems, poisoning, tapeworms, bladder and kidney troubles, body soreness, etc. Also, some species have shown antioxidant, anti-inflammatory, and hypoglycemic activities (39). There have been phytochemical investigations regarding the chemical composition of the *Eryngium* genus. Those studies have been analyzing the leaves and the roots of this species and the great majority of the genus have shown the presence of essential oils, acetylenes, coumarins, saponins, flavonoids, and rosmarinic acid derivatives mainly (40).

In México, the genus *Eryngium* is mostly well-known as “Hierba del sapo” or “Frog grass” indistinctly of the different species there exist as they share similar characteristics, only being distinguishable among “hierberos” by the location or state that they grow. This plant is popularly used for treating T2D and dyslipidemias. Traditionally is consumed as an infusion (aqueous extract), and it has been calculated that approximately 20 g of this plant is being consumed by a person (using an approximation that a person weighs around 70 kg) (41). *Eryngium carlinae* is perhaps the most studied species as there are numerous papers that highlight its importance, its medicinal properties, its main characteristics. It is considered that indigenous communities are the ones that are much more familiar with the knowledge, use, and recognition of medicinal herbs as they live in rural areas where medical attention is scarce (42).

### *Eryngium* Phytocomponents and Their Medicinal Properties

The main components of *Eryngium* spp. that can be found in the leaves are caffeic, chlorogenic, and rosmarinic acids. These phenolic acids have reports of improving glucose metabolism in mice. Caffeic acid reduces the blood glucose levels and glycated hemoglobin (HbA1c) in insulin-resistant mice, through the inhibition of phosphoenolpyruvate carboxykinase and glucose 6-phosphatase (G6Pase), a reduction in expression of hepatic glucose transporter 2 and augmentation of adipocyte transporter 4 (43). Rosmarinic acid reduces blood glucose levels in HbA1c and Homeostatic Model Assessment Insulin Resistance (HOMA-IR) significantly in those rats that were induced to diabetes with a high-fat diet and streptozotocin, which is a compound that has preferential toxicity toward pancreatic β cells (41). Also, this phenolic acid reduced the increased enzymatic activity of G6Pase and fructose 1, 6-bisphosphatase (FBPase) and reestablished the impaired activity of enzymes related to glucose oxidation and glycogen synthesis in that particular diabetic model that was analyzed (41). Furthermore, it has been seen that the terpenes and sesquiterpenes like β-farnese, β-pinene, and calamenene that are present in the leaves of *Eryngium carlinae* have a protective effect by reducing the oxidizing damage in brain, kidney, and liver of diabetic rats. This effect is related to the ROS that are produced when oxidative stress happens when there is an increase of glucose because of diabetes (9). Also, in another study with Streptozotocin diabetic induced mice, it was observed that the ethanolic extract of *E. carlinae* reduced the amount of creatinine, uric acid, total cholesterol and triglycerides levels in diabetic mice in comparison with healthy mice, thus improving renal function (44). In Table 1 there are described some of the main components of *Eryngium* spp. as well their location in the plant and the registered species that contain such components.

**Table 1:** Phytochemical compounds found in aerial parts of *Eryngium* spp.

**Reported plant**	**Compound**	**Reference**
**Acetylenes**		
*Eryngium campestre, Eryngium carlinae, Eryngium caeruleum, Eryngium macrocalyx, Eryngium dichotomum, Eryngirum creticum*	D-Mannitol	(45)
*Eryngium dichotomum*	D-furanose	
*Eryngium agavifolium*	Hexaecanoic acid	
**Caffeic acid esther**		
*Eryngium bourgatii, Eryngium foetidum*	Rosmarinic acid	(45, 46)
**Cinnamic acids**		
*Eryngium bourgatii*	Ferulic acid	(45)
	Deltoin	
**Coumarins**		
*Eryngium creticum*	Deltoin	(45)
*Eryngium bieberstertarium*	Bergaptin	
*Eryngium campestre*	Aegelinol benzoate	
	Grandivittin	
**Flavonoids**		
*Eryngium bourgatii, Eryngium foetidum, Eryngium campestre, Eryngium planum*	Kaempferol dihexoside	(45, 46)
*Eryngium creticum*	Quercetin	(45)
*Eryngium campestre, Eryngium octophyllum*	Rutin	(45)
**Phenols**		
*Eryngium bourgatii, Eryngium carlinae*	Caffeic acid	(45)
*Eryngium bourgatii, Eryngium alpinum*	Chlorogenic acid	
*Eryngium carlinae*	Gallic Acid	(47)
	Ellagic acid	
**Saponins**		
*Eryngium yuccifolium*	Saniculasaponin III	(45)
	Eryngiosides A-L	
*Eryngium carlinae*	Campesteryl-β-D-glucopyranoside	(46)
	Sitosteril-β-D-glucopyranoside	
**Sequiterpenes**		
*Eryngium giganteum*	Trans-β-farnesene	(45)
**Steroids**		
*Eryngium foetidum*	β-Sitosterol	(45, 46)
	Stigmasterol	
*Eryngium foetidum, Eryngium agavifolium*	Brassicasterol	
*Eryngium carlinae*	Δ5-Avenastarol	(47)
	β-Campesterol	
**Tripernoids**		
*Eryngium bromeliifolium*	Betulinic acid	(45)
*Eryngium macrocalyx*	Oleanolic acid	

### *Eryngium* spp. and Lipid Control

**Figure 3:**
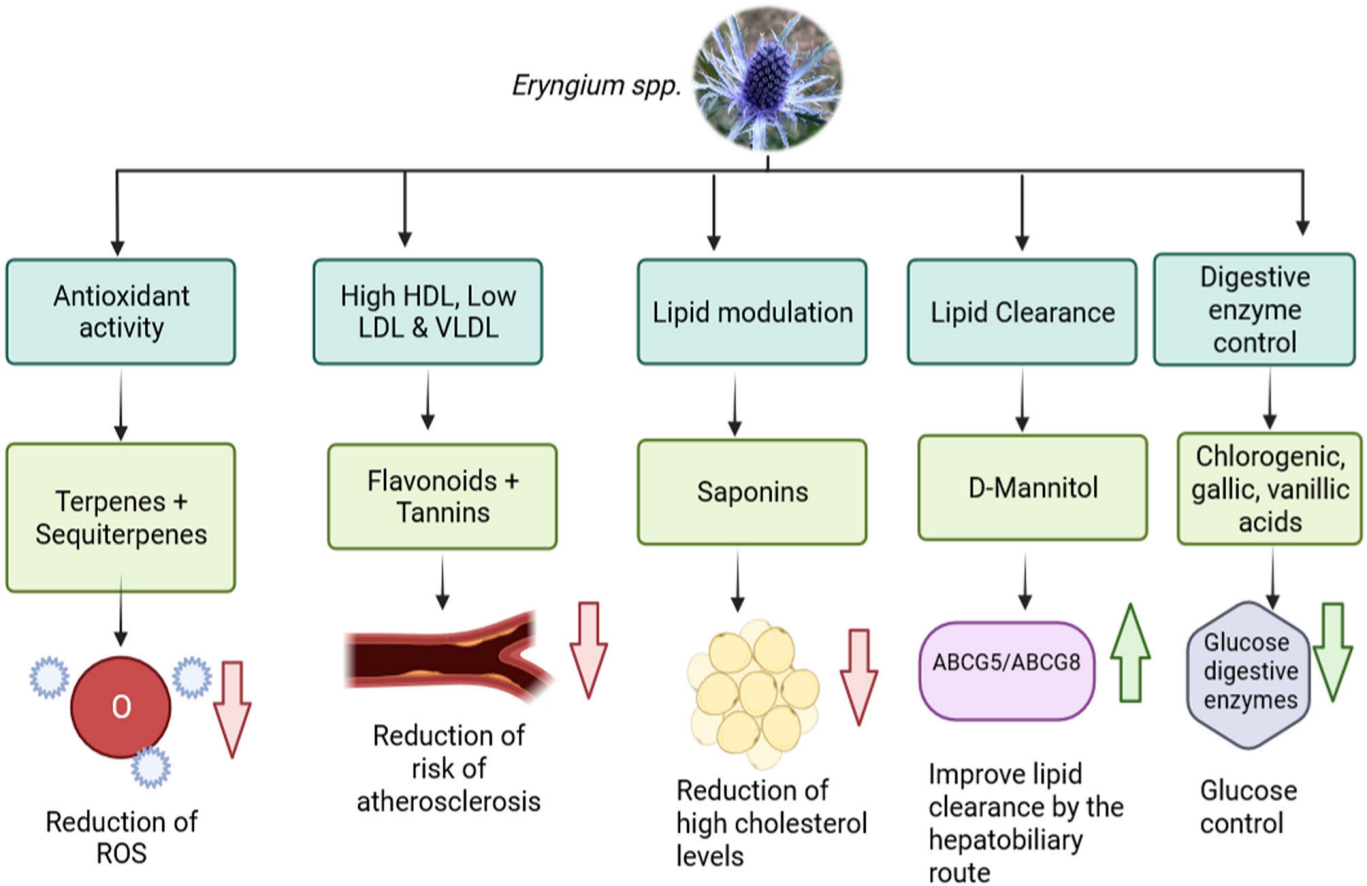
Benefits of the phytocomponents of *Eryngium* spp. with lipid and glucose control (ROS, Reactive Oxygen Species; NPC1L1, Niemann-Pick C1-Like 1; ABCG5, ATP-Binding Cassette Subfamily G Member 5; ABCG8, ATP-Binding Cassette Subfamily G Member 8) (Created in BioRender).

Other components of *E. carlinae* are triterpene saponins, which played an important role in dyslipidemia by reducing the serum concentrations of lipids in mice (48). In another study, a group of rats were induced into diabetes and were given an ethanolic extract of *E. carlinae* and Atorvastatin both, separately and together, for 40 days (44). This study revealed that there was no effect on glucose levels, but it reduced total cholesterol and triglycerides levels. Furthermore, it was observed that *E. carlinae* extract intake can increase HDL levels and reduce the levels of LDL and VLDL, the atherogenic index, which prevents the risk of suffering a cardiovascular event. Another study, showed that *E. carlinae* extract reversed the phenomenon of lipid peroxidation in diabetic mice, reducing the effect of oxidation stress that diabetes can cause due to insulin resistance (9). Regarding the transporters ABCG5/ABCG8, the consumption of *E. carlinae* in diabetic people increases the levels of both transporters, which would improve the disposal of cholesterol in patients, but more research is needed in this area (49). As discussed earlier, the aerial parts of *Eryngium* are normally consumed as an infusion to obtain the benefits of the plants. However, a recent study made with streptozotocin/nicotinamide- induced type 2 diabetes rats were given the ethanolic extract of *E. billardieri* obtained from the roots, revealed that the content of flavonoids and tannins of the extract increased the levels of HDL and reduced the levels of VLDL, LDL. And also it was observed that the saponins present in the extract had the ability to modulate lipid profiles by suppressing cholesterol luminal absorption, and increasing cholesterol secretion through the biliary excretion (50), this study may imply that the whole plant has hypolipidemic effects and it is not only reduced to the aerial part, though more investigation may be needed in this area. Another study evaluated the antioxidative power of *E. maritimum L*. of the aerial parts of the plant, which was consistent with other *Eryngium* species. The novelty of the study revealed that the tinctures of the aerial parts of *E. maritimum* also had the capacity of inhibiting dietary carbohydrate digestive enzymes, meaning this could be beneficial to control glucose levels and aid in managing T2D (51). These benefits can be summarized in Figure 3.

### *Eryngium* spp. and Diabetes

In Mexico, *Eryngium* spp. has traditionally been used to treat diabetes. Particularly in the form of decoctions of the aerial parts of the plant, however there are very few studies that evaluate the effects of its consumption. However, in a study conducted by Noriega-Cisneros where diabetes was induced in rats by intraperitoneal administration of Streptozotocin, the ethanolic extract of *E. carlinae* was administered and it was observed that diabetic rats reduced their creatinine level, thus improving their renal function. A reduction in uric acid levels was also observed, slowing the progression of diabetes. Another improvement was the reduction of triglyceride levels, so it was possible to see that the *E. carlinae* extract could be used as an adjuvant in the treatment of diabetes (52). It has previously been mentioned that one of the main problems in diabetes is the oxidative stress caused by ROS, which can lead to cardiac complications or lipotoxicity. The hexane extract of *E. carlinae* was administered to rats induced with diabetes and it was observed that it increased Nitrogen Oxide (NO), that is, it increased the bioavailability of NO, leading to an endothelial protective effect and the reduction of oxidative stress (53). Regarding the nephroprotective effect that *Eryngium* spp. there have been some studies on this problem. In the study provided by Pérez-Ramírez (47), mice induced with diabetes took the decoction of *E.carlinae* and the reduction of renal oxidative stress was observed, as well as the reduction of pro-inflammatory proteins such as Activin A, Fas ligand, ICAM-1, IFN-γ, IL-1β, IL-1R6, IL-13,leptin, RAGE, TNF-α; among other proteins, thus reducing the inflammation. The authors justify this property due to the polyphenols, saponins and phytosterols that are present in *E. carlinae* (47). In another study, the combination of *E. billardieri* and Metformin® was assessed, results showed that *Eryngium* extract with its antioxidant properties, can improve the injury of renal tubular cell damage caused by oxidative stress in diabetic rats. While Metformin inhibits functional and structural damage of renal cells and hypoxia by using AMPK activation, this combination improved oxidative stress conditions and prevented diabetic renal injury (5). In a more recent study provided by del Cetto (54), author addressed the problem of impaired hepatic glucose production during gluconeogenesis, which is the main source of fasting hyperglycemia, making it one of the contributors to postprandial hyperglycemia in diabetic patients. The main enzymes in the pathway are G6Pase and FBPase. The ethanolic extract of *E. longifolium* was evaluated and it was observed that it inhibited the activity of G6Pase by 75% and FBPase was inhibited 100% with a concentration of 5,000 μg/mL. The presence of rosmarinic and chlorogenic acids, which *E. longifolium* have as major compounds, were responsible for inhibiting such enzymes, since chlorogenic acid is a weak inhibitor of α-glucosidases and rosmarinic acid has a significant reduction power over G6Pase and FBPase, thus controlling the overproduction of glucose in diabetes (54). The possibility has been raised that the efficiency of the process is due to the solubility of active polar organic compounds in it, since most of the active polar compounds tend to be extracted with chloroform and ethyl acetate, leaving a very small proportion of remaining compounds in the last fraction (28).

### Extraction Methods for Screening of Phytochemicals From *Eryngium* spp.

The extraction process and subsequent recovery of phytochemicals from plant materials such as *Eryngium* spp., are generally affected by the type of extraction and its conditions such as type of solvent and polarity, temperature, pressure and extraction time. The extraction of phenolic compounds from *Eryngium* spp. it is generally performed using conventional solid-liquid techniques. For instance, maceration with hot water at 40°C (55), and organic solvents like methanol (56) or ethanol (57) are the most common extraction methods. However, these traditional methods had low extraction yields, poor selectivity and involves the use of large amounts of organic solvents. By these means, making it an environmentally unfriendly method, jeopardizing their beneficial application (58). For these reasons, novel technologies with environmental low impact have been studied, such as cavitation, ultrasound assisted, microwave assisted, and supercritical fluid extractions. For example, the effect of acoustic cavitation on the separation of bioactive compounds from *E. caucasicum* was assessed (59). In this study, the effect of acoustic cavitation conditions and two levels of temperatures (30–60°C) and ultrasonic power levels of (50–150 W) were addressed, in which the sample with ethanol was subjected to 60 min of sonication. The results showed that the highest extraction yield was achieved at an ultrasonic power of 112.10 W, an extraction temperature of 50 °C and 33.53 minutes of sonication. Also, the total phenolic content of this extract was 64.00 ± 0.13 mg GAE/g, and was identified that gallic acid, chlorogenic acid, *p-* coumaric acid, ferulic acid were the main components present in the sample. Another methodology explored for the extraction of bioactive compounds from this genus is assisted ultrasound. Filho et al. (60) obtained phenolic compounds from *E. foetidum* using a 70% ethanol solution and 70% of maximum amplitude of ultrasound (20 kHz) for 10 min at 20°C (60). The total phenolic content of this extract was 10.8 ± 0.56 mg GAE/g, and was identified that flavonoids (9.12 ± 0.32 mg quercetin g^−1^) and anthocyanins (0.21 ± 0.02 mg cyanidin−3-glucoside g^−1^) were the main components present in the sample. In a similar study, chlorophyll (30.0 ± 2.30 μg mL^−1^) and saponins (84.01 ± 1.20 mg diosgenin g^−1^) were obtained using ultrasound with higher extraction yields compared to conventional solid-liquid technique (60). Also, G. Paun (61) evaluated the efficacy of assisted ultrasound in *E. planum* materials, using a sonicator water bath at 35 kHz for 90 min, in order to obtain polyphenolic rich extracts (61). The ultrasound *E. planum* extracts contained mainly flavonoids especially rutin and isoquercitrin, with ursolic acid at a concentration of 76.77 μg/mL to 158.04 μg/mL. Furthermore, the supercritical fluid extraction was also studied in dried *E. maritimum* at 47°C and pressure of 300 bars with 4% of ethanol as co-solvent (62). The supercritical CO_2_ extraction allowed a selective extraction of compounds with higher biological activities such as antioxidant, antimicrobial, anti-collagenase and antityrosinase activities. Finally, the combination of extraction technologies can favor the obtaining yield of bioactive compounds. For instance, the combination of ultrasound with microwave assisted extraction, increased the total phenolic content from *E. maritimum* extracts (62). Microwave conditions at 25 kHz for 1h 30, with 900 W for 45 min in combination with ultrasound, significantly increase the extraction of total phenolic compounds. In conclusion, selective metabolites obtained from *Eryngium* spp., through novel technologies allowed to correlate chemical diversity with bioactivity and increased the yield of bioactive.

### Future Trends

In the new global economy, ethnopharmacology has become a central issue for reliable alternatives toward metabolic syndrome conditions. The present review raises the possibility to use traditional medicinal herbs as important sources to obtain bioactive compounds that may be used as adjuvants in the treatment of these diseases. *Eryngium* spp. has been studied due to their beneficial properties mainly to treat diabetes, dyslipidemia, blood pressure, and digestive problems. However, the most interesting finding was the lack of full characterization of *Eryngium* species to further understand and validate the mechanism of action of their bioactive components. As mentioned in the literature review, the metabolic syndrome involves interconnected physiological, biochemical, clinical, and metabolic factors, thus, further controlled studies must be performed in other to determine the downregulation of the metabolites involved. For this matter, the cellular mechanism of interactions must be addressed to understand the biological implications of *Eryngium* species. This will lead to improved use of traditional plants through the performance of preclinical studies. Future investigations on phytochemicals health care applications from these species should include their potential toxicity and their degradation in the gastrointestinal lumen before being absorbed. Special attention should be paid to the effect of the digestive conditions. Once the safety, bioaccessibility, and bioavailability of the phytochemicals are assessed it is crucial to elucidate new delivery systems of bioactive compounds to improve their use at the industry that enables targeted products in health care. Further investigation and experimentation considering circular economy processing is strongly recommended. As it was discussed before that the roots of the plant may be as effective as the aerial part of the plant this would allow the use of the complete plant and thus reduce waste, but also this could be an opportunity to apply synthetic biology to produce the necessary antioxidants to prevent lipid toxicity. This could allow the potential application of phytochemicals toward metabolic syndrome impairments.

## Author Contributions

EP-M, MM-Á, and DG-F contributed to conception and design of the study. EP-M and MM-Á organized the database. EP-M wrote the first draft of the manuscript. EP-M, MM-Á, MA-R, and DG-F wrote sections of the manuscript. All authors contributed to manuscript revision, read, and approved the submitted version.

## Conflict of Interest

The authors declare that the research was conducted in the absence of any commercial or financial relationships that could be construed as a potential conflict of interest.

## Publisher's Note

All claims expressed in this article are solely those of the authors and do not necessarily represent those of their affiliated organizations, or those of the publisher, the editors and the reviewers. Any product that may be evaluated in this article, or claim that may be made by its manufacturer, is not guaranteed or endorsed by the publisher.
